# Annual acknowledgement of manuscript reviewers

**DOI:** 10.1186/1472-6831-13-14

**Published:** 2013-03-13

**Authors:** Christopher Foote

**Affiliations:** 1 BMC Series, BioMed Central, 236 Grays Inn Road, London WC1X 8HB, UK

## Abstract

**Contributing reviewers:**

The editors of BMC Oral Health would like to thank all the reviewers who have contributed to the journal in volume 12 (2012).

## 

Kavita Ahluwalia

USA

Rahena Akhter

Bangladesh

Jolanta Aleksejuniene

Canada

Paul Allison

Canada

Jose M Almerich

Spain

Mohammad Ahmad Al-Omari

Jordan

Flávio Alves

Brazil

Najith Amarasena

Australia

Jose Leopoldo Ferreira Antunes

Brazil

Antonio Antunes

Brazil

Thiago Ardenghi

Brazil

Eiichiro Ariji

Japan

Wolfgang H. Arnold

Germany

Paul Ashley

UK

Anne Nordrehaug Astrom

Norway

Paul Aveyard

UK

Ross Bailie

Australia

Howard Bailit

USA

Isabelle Bailleul-Forestier

France

Ben Balevi

Canada

Christophe Bedos

Canada

Lin Bin

China

Nabil Bissada

USA

Lars Bjorkman

Norway

Henk Brand

Netherlands

Jonathan Broadbent

New Zealand

Paul Brocklehurst

UK

Josef Bruers

Netherlands

Heather Buchanan

UK

Girvan Burnside

UK

Maria Grazia Cagetti

Italy

Guglielmo Campus

Italy

Marta Ana Carballo

Argentina

Alessandro Cavalcanti

Brazil

Asja Celebic

Croatia

James Chambers

USA

Luis Chavez de Paz

USA

Ivor Chestnutt

UK

Donald Chi

USA

Lin-P'ing Choo-Smith

Canada

Chun-Hung Chu

Hong Kong

Peter Cleaton-Jones

South Africa

Dilsah Cogulu

Turkey

Rita Cordeiro

Brazil

Sybil Crawford

USA

Jaime Cury

Brazil

Brian W Darvell

Kuwait

Roeland De Moor

Belgium

Cornelis de Putter

Netherlands

Michael de Vrese

Germany

Sandra Decker

USA

Deirdre Devine

UK

Jennifer DeVoe

USA

Michele Diniz

Brazil

Marcia Ditmyer

USA

Kimon Divaris

USA

Virginia Dodd

USA

Mark Donaldson

USA

Bernadette Drummond

New Zealand

Alexandrina Lizica Dumitrescu

Norway

Cristiane Duque

Brazil

Lars Eliasson

Sweden

Camile Farah

Australia

Ali Farahani

UK

Efigênia Ferreira

Brazil

BA Finegan

Canada

Morenike Folayan

Nigeria

Lyndie Foster Page

New Zealand

Kenji Fueki

Japan

Peter Gaengler

Germany

Jenny Gallagher

UK

Marina Gallottini

Brazil

Maria Beatriz Gaviao

Brazil

Tarik Gheit

France

Jason Goodchild

USA

Barbara Greenberg

USA

Gianpaolo Guzzi

Italy

Magnus Hakeberg

Sweden

Mamata Hebbal

India

Martine Hennequin

France

Dorthe Holst

Norway

Matthew Hopcraft

Australia

Alice Horowitz

USA

Sarah Horton

USA

Colleen Huebner

USA

Emi Inada

Japan

Mohamed Jaber

United Arab Emirates

Anahita Jablonski-Momeni

Germany

Sok Janket

USA

Lysle Johnston

USA

Kota Katanoda

Japan

Katerina Kavvadia

Greece

Yumiko Kawashita

Japan

Nazeer Khan

Pakistan

Nicky Kilpatrick

Australia

Karl Kingsley

USA

Julia Kravchenko

USA

Jung Hye Kwon

South Korea

Binnaz Leblebicioglu

USA

Jessica Lee

USA

Roos Leroy

Belgium

Steven Levy

USA

Edward Lo

Hong Kong

Vuokko Loimaranta

Finland

Mark Macek

USA

Melinda Madlena

Hungary

Maria-Cristina Manzanares-Céspedes

Spain

Zoe Marshman

UK

Michael Martin

USA

Gerardo Maupome

USA

Colman McGrath

Hong Kong

John McIntyre

Australia

Fausto Mendes

Brazil

Marcelo Meneghim

Brazil

Lisa Metsch

USA

Theodorus Mettes

Netherlands

Peter Milgrom

USA

David Mitchell

UK

Bella Monse

Philippines

Pranab Mukherjee

USA

Paulo Nadanovsky

Brazil

Munetaka Naitoh

Japan

Kazuhiko Nakano

Japan

Cassio Nascimento

Brazil

Ian Needleman

UK

Man Wai Ng

USA

Pasquale Niscola

Italy

Gen Nishimura

Japan

Ryota Nomura

Japan

Eyitope Ogunbodede

Nigeria

Won-suk Oh

USA

Kerstin Öhrn

Sweden

Luciana Oliveira

Brazil

Chukwudi Ochi Onyeaso

Nigeria

Germano Orrù

Italy

Richard Osborne

Australia

Anna-Lena Ostberg

Sweden

Constantine Oulis

Greece

Saul Paiva

Brazil

William Papaioannou

Greece

Antonio Carlos Pereira

Brazil

Chaiana Piovesan

Brazil

Kelly Polido Kaneshiro Olympio

Brazil

Alexander Ponizovsky

Israel

Pier Francesco Porciani

Italy

Archana Pradhan

Australia

Velia Ramìrez Amador

USA

Maria Letícia Ramos-Jorge

Brazil

Lia Rimondini

Italy

Jonas Rodrigues

Brazil

Stephen Rosenstiel

USA

Monique Rothan-Tondeur

France

R Gary Rozier

USA

Charles Rwenyonyi

Uganda

Wael Sabbah

UK

Matteo Saccucci

Italy

Toshiyuki Saito

Japan

Atsushi Saito

Japan

Silvia Sales-Peres

Brazil

Linda Scheirton

USA

Stefan Schreiber

Germany

Robert Schroth

Canada

Lai-Chu See

Taiwan

Jadbinder Seehra

UK

Juan Jose Segura-Egea

Spain

Gary Slade

Australia

Grace Smith

USA

Oyinkansola Sofola

Nigeria

Tewarit Somkotra

Thailand

Armando Soto-Rojas

USA

Catriona Steele

Canada

Charles Streckfus

USA

Stefan Stuebinger

Switzerland

Ruth Tachezy

Czech Republic

Santosh Kumar Tadakamadla

India

William Murray Thomson

New Zealand

Asli Topaloglu Ak

Turkey

Jefferson Traebert

Brazil

Paula Trevilatto

UK

Georgios Tsakos

UK

Yu-Kang Tu

UK

Stephanie Tubert-Jeannin

France

Eduard Valmaseda-Castellón

Spain

Frank van de Veerdonk

Netherlands

Wil van der Sanden

Netherlands

Cor van Loveren

Netherlands

Miira M Vehkalahti

Finland

Mario Vettore

Brazil

Jorma Virtanen

Finland

Arjan Vissink

Netherlands

John Warren

USA

Richard Watt

UK

Sandy Whitelaw

UK

Ephraim Winocur

Israel

Manabu Yanagita

Japan

Cynthia Yiu

Hong Kong

## Pre-publication history

The pre-publication history for this paper can be accessed here:

http://www.biomedcentral.com/1472-6831/13/14/prepub

